# Standardization of Animal Models and Techniques for Platelet-Rich Fibrin Production: A Narrative Review and Guideline

**DOI:** 10.3390/bioengineering10040482

**Published:** 2023-04-17

**Authors:** Carlos Fernando Mourão, Adam Lowenstein, Rafael Coutinho Mello-Machado, Shahram Ghanaati, Nelson Pinto, Tomoyuki Kawase, Gutemberg Gomes Alves, Michel Reis Messora

**Affiliations:** 1 Department of Periodontology, Division of Dental Research Administration, Tufts University School of Dental Medicine, Boston, MA 02111, USA; 2 Department of Implant Dentistry, Universidade Iguaçu, Nova Iguaçu 26260-045, Brazil; 3 Frankfurt Oral Regenerative Medicine, Clinic for Maxillofacial and Plastic Surgery, Johann Wolfgang Goethe University, 60596 Frankfurt Am Main, Germany; 4 Department of Periodontics and Implant Dentistry, University of the Andes, Santiago 12455, Chile; 5 Division of Oral Bioengineering, Niigata University Graduate School of Medical and Dental Sciences, Niigata 951-8514, Japan; 6 Clinical Research Unit, Antonio Pedro Hospital, Fluminense Federal University, Niterói 24033-900, Brazil; 7 Department of Oral and Maxillofacial Surgery and Periodontology, School of Dentistry of Ribeirao Preto, University of Sao Paulo, Ribeirao Preto 14040-904, Brazil

**Keywords:** platelet-rich fibrin, animal models, experimental research, guideline

## Abstract

Experimental research is critical for advancing medical knowledge and enhancing patient outcomes, including in vitro and in vivo preclinical assessments. Platelet-rich fibrin (PRF) is a blood by-product that has garnered attention in the medical and dental fields due to its potential for tissue regeneration and wound healing. Animal models, such as rabbits and rats, have been used to produce PRF and examine its properties and applications. PRF has demonstrated potential in the dental and medical fields for reducing inflammation, promoting tissue repair, and accelerating wound healing. This narrative review aims to compare existing evidence and provide guidelines for PRF animal research, emphasizing the importance of standardizing animal models, following ethical considerations, and maintaining transparency and accountability. The authors highlight the necessity to use the correct relative centrifugal force (RCF), standardize centrifugal calibration, and report detailed information about blood collection and centrifuge parameters for reproducible results. Standardizing animal models and techniques is crucial for narrowing the gap between laboratory research and clinical applications, ultimately enhancing the translation of findings from bench to bedside.

## 1. Introduction

Translational research is a critical process that involves the translation of basic scientific discoveries into practical applications that benefit human health [1]. It consists of bridging the gap between basic research and clinical practice by using experimental studies to develop new techniques and procedures in the medical field. Experimental studies play a crucial role in the development of new medical techniques and procedures, as they provide the necessary evidence to support the effectiveness and safety of these innovations. For example, clinical trials are essential to testing the efficacy of new treatments and drugs before they are approved for use in patients. Advancing medical knowledge and developing new treatments that improve patient outcomes would be impossible without experimental studies, including important results derived from in vitro and in vivo preclinical assessments. The presence of experimental research in translational research is a vital step in ensuring that scientific discoveries are translated into practical applications that improve human health [2,3].

In the past two decades, platelet-rich fibrin (PRF) has become increasingly popular in medicine and dentistry for its potential use in wound healing and tissue regeneration [4,5,6,7,8]. However, PRF’s development deviated from the typical research process as it was first used in humans before being studied in vitro and with animal models [9]. This unconventional approach was taken to enhance the research and gain a better understanding of the blood by-product. This sequence of application was likely due to the urgent need for effective treatments in human patients, leading to the rapid adoption of PRF in clinical practice. As more knowledge and experience were gained, researchers began to study PRF in vitro and in animal models to better understand its mechanisms of action and optimize its use [10,11]. Such studies have yielded valuable insights into the properties and potential applications of PRF, paving the way for further translational research to develop new techniques and procedures [12,13,14,15].

In order to improve the techniques and discover new clinical applications, animal models, such as rabbits [16,17,18,19,20] and rats [21,22,23], have been used to produce PRF and investigate its properties and potential applications. The high concentrations of platelets, growth factors, and other bioactive molecules make PRF useful in promoting tissue regeneration and enhancing bone growth [11]. In the dental field, the blood by-product has been used in various procedures to reduce inflammation, accelerate wound healing, and improve outcomes. In the medical field, PRF has been used to treat chronic wounds, burns, and musculoskeletal injuries, with promising results in enhancing tissue repair and reducing inflammation [24,25].

Fibrin research has shown that fibrin clots in mammals such as mice, rats, and rabbits can exhibit different fiber diameters and clot densities compared to human clots. These differences have been considered when using animal models to study blood clotting and related disorders in humans, as they can inform therapeutic development and enhance our understanding of blood clotting mechanisms in general [26,27].

Compared to platelet-rich plasma (PRP), PRF does not necessitate the addition of anticoagulants or other external factors during its preparation. This simplifies the process and potentially mitigates the risk of adverse effects related to additives [27]. Nevertheless, the anticoagulants in PRP can aid researchers in acquiring the appropriate amount of this blood derivative for use in animal models, giving them more time during the blood draw.

The major concern about PRF’s production using animal models is the necessity to have fast blood collection, which is essential in producing high-quality PRF as it minimizes the risk of clotting and ensures that the concentration of platelets and other bioactive molecules remains high. However, in some animal species, such as rabbits and rats, blood coagulates quickly, making it necessary to collect blood rapidly to avoid clotting. In addition, tubes with clot activators, such as silica, can also aid in blood coagulation and make PRF’s production difficult. In addition, access to appropriate vessels (veins or arteries) from animals can be challenging, and the amount of blood that can be obtained may be limited. In this context, PRF has been investigated through very diverse animal models with a great heterogeneity of methodologies, with little help available for the choice of the best protocol for the production of this blood byproduct.

The aim of this narrative review is to compare the available evidence and provide a helpful guideline for animal research on PRF. The guideline aims to assist researchers in avoiding unnecessary loss of time and taking the lives of animals during the study process. It is essential to emphasize the importance of animal welfare and ethical considerations in research, and this guideline serves to clarify and guide future studies in this area.

## 2. Definitions and Search Process

Experimental studies aim to investigate the properties and potential applications of PRF. For this narrative review, the authors conducted a literature search in PubMed/MEDLINE databases for experimental studies involving the most common methods of blood collection used in animal studies for PRF production.

## 3. Overview of Animal Research

Animal care is a crucial aspect of any animal study, as researchers are responsible for ensuring that their subjects are treated humanely and with the utmost respect. This consists of providing appropriate housing, nutrition, and care to minimize any potential harm that may be inflicted on them during the study. In addition, by establishing an ethical committee for animal studies, researchers can ensure that all aspects of the study are reviewed by an independent body to ensure compliance with ethical standards and regulations [28].

In addition to ethical oversight, guidelines are essential in any animal study. The ARRIVE guidelines were developed to provide a checklist of information that should be included in reports of in vivo experiments [29]. These guidelines help to ensure transparency and consistency in reporting by providing a standardized format that contains information about animal welfare, housing, and health status. These guidelines also ensure that researchers provide sufficient detail on their experimental design, methods, and results, allowing others to reproduce the study if necessary. They are of special interest in platelet concentrate research as these products depend on the content of several growth factors and inflammatory mediators whose production and release may be affected by different biological factors, health status, and stress.

Similarly, the PREPARE guidelines were developed to provide a framework for the planning phase of animal studies [30]. By considering all the factors that may influence the study’s outcome during the planning phase, researchers can ensure their study is well-designed and scientifically rigorous. This includes considering factors such as the choice of animal model, sample size, experimental design, and statistical analysis.

The importance of these guidelines cannot be overstated, as they provide clear and concise instructions for the ethical use of animals in research. They promote transparency, consistency, and accountability, ultimately enhancing the research findings’ credibility.

Sample size calculation is another critical aspect of studies involving animals [31,32]. By determining the minimum number of animals required to achieve statistical power, researchers can ensure that their research is adequately powered to detect any effects of the intervention or treatment being tested. Furthermore, sample size calculations consider factors such as the expected effect size, variability, and significance level to ensure the study is well-designed and scientifically rigorous. Adequate sample sizes also help to minimize the number of animals used in the study, reducing any potential harm that may be inflicted on them.

## 4. Considerations on Blood Collection

When collecting blood from animals for research, it is essential to prioritize the safety and well-being of the animals, which requires adhering to similar guidelines as those followed in human blood donation. One fundamental recommendation is the “10% rule”, which states that the amount of blood drawn should not exceed 10% of the animal’s total blood volume [33,34]. For instance, a 2 kg rabbit has an average blood volume of approximately 55–70 mL per kilogram of body weight, translating to a total blood volume of around 110–140 mL. Hence, based on the 10% rule, a maximum of 11–14 mL of blood can be safely collected from this rabbit to avoid any adverse effects. By following these guidelines, researchers can collect blood from animals safely and humanely in a manner that does not jeopardize their health.

Similarly, when collecting blood from rats, it is essential to use proper techniques and equipment. The amount of blood that can be safely collected from a rat depends on several factors, including body weight, age, and strain. As a general guideline, the amount of blood that can be safely drawn from a rat should not exceed 10% of its total blood volume. For example, a 250-g rat has a total blood volume of approximately 15–20 mL, so a maximum of 1.5–2 mL of blood can be safely collected from this rat.

In addition, ensuring safe and adequate blood collection involves adhering to proper techniques and equipment, and selecting the appropriate vessel and blood flow for the study type. The choice of vessel and blood flow depends on the desired blood by-product and its specific requirements. For instance, in PRP studies [35,36,37,38,39], anticoagulants are used, such as sodium citrate, which do not require fast blood flow to the collection tube. Conversely, for PRF studies, a vessel with rapid blood flow is necessary due to the absence of anticoagulants and the use of clot activators such as silica, making fast blood obtention essential to produce this blood concentrate.

## 5. Venipuncture of Rats

Blood collection in rats is a common procedure in many research studies that require blood samples for analysis (e.g., cytokines and growth factors) or for blood concentrate production [40,41,42,43,44,45,46]. There are several anatomical sites from where blood can be collected from rats; the most common are the tail vein, intracardiac function, and orbital sinus (Table 1).

Blood collection from the tail vein is relatively easy and minimally invasive, making it a preferred option for many researchers. The rat’s tail is first warmed with a heat lamp to dilate the veins, and sometimes a small incision is made to collect the blood. This method is used for collecting small amounts of blood and is suitable for tests that require minimal manipulation of the blood sample.

**Figure 1:**
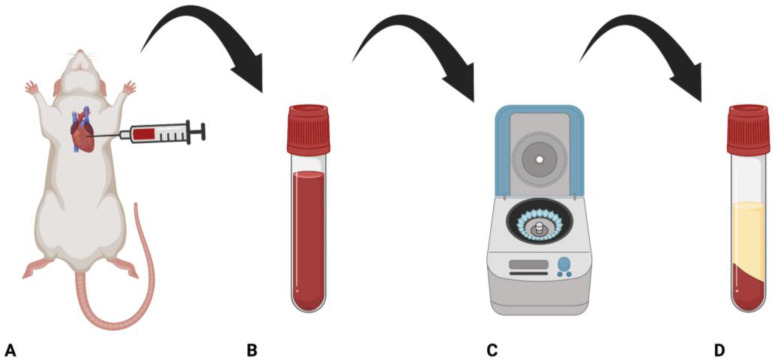
(**A**) Intracardiac puncture in the rat’s heart using a syringe with a needle ranging between 18 to 23G; (**B**) Blood placed in tube (without additives); (**C**) The tube is then quickly placed in the centrifuge, following protocols for PRF production, rpm, time, and relative centrifugal force (RCF); (**D**) PRF is produced after following the previous steps.

Intracardiac is a more invasive blood collection method involving accessing the heart directly. It requires surgical skills and experience and should be performed under anesthesia (Figure 1). This method allows for the collection of larger volumes of blood and is more suitable for tests that require more extensive manipulation of the blood sample. Therefore, intracardiac should be the first area of election to produce the PRF from the rats.

**Figure 2:**
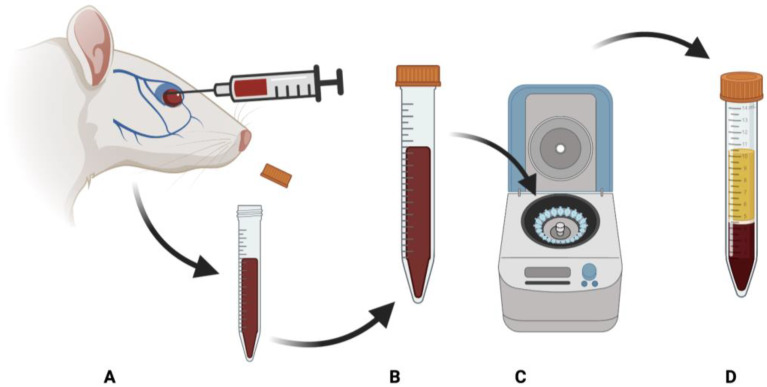
(**A**) Orbital sinus puncture in the rat’s orbital area using a syringe with a needle ranging between 21–23G; (**B**) Blood placed in a tube/falcon (without additives); (**C**) The tube is then quickly placed in the centrifuge, following protocols for PRF production, rpm, time, and RCF; (**D**) PRF is produced after following the previous steps.

Table 1 shows another option for blood collection in rats, the orbital sinus that involves accessing the veins located behind the rat’s eyes (Figure 2). Although this blood collection method is simple to carry out and is applicable for tests, with minimal manipulation of blood samples and smaller volumes, it may be ineffective in situations where low blood flow is an issue. Insufficient blood flow, which can result from certain medical conditions or injuries, may restrict the amount of blood obtained using this method. Thus, in such circumstances, alternative blood collection methods such as venous or arterial puncture may need to be used to obtain the required amount of blood for testing. These observations provide a comprehensive view of the advantages and limitations of this blood collection method and emphasize the significance of considering alternative techniques when blood flow is reduced.

Once the blood sample is collected for PRF production, it is essential to understand that the coagulation time for rat blood is around 2–5 min, which is faster than the human coagulation time of 5–10 min [65,66,67,68,69,70]. Considering this information, the researcher needs a fast blood collection from a vessel with satisfactory blood flow. After that, the tube must go quickly to the centrifugation process.

In terms of the number of cells in the blood, rats have a higher number of red blood cells and a lower number of white blood cells compared to humans. It is important to note that the platelet count in rats can vary widely depending on various factors such as age, sex, strain or breed, and health status. The platelet count in rats can range between 600,000 and 1,500,000. Therefore, it is recommended to consider these factors when interpreting the platelet count in rats for PRF production. Additionally, the quantity of cytokines and growth factors in rat blood varies based on these [71,72,73]. Therefore, it is essential to consider these factors when conducting research using rat blood samples and comparing data from different studies in the literature.

## 6. Venipuncture of Rabbits

Rabbits are another animal model used to study different types of blood concentrates [74,75,76,77,78,79]. Considering the animal’s anatomy, there are several areas where blood can be collected from rabbits. The most common areas found in studies involving PRF include the ear vein, intracardiac, and jugular (Table 2).

**Figure 3:**
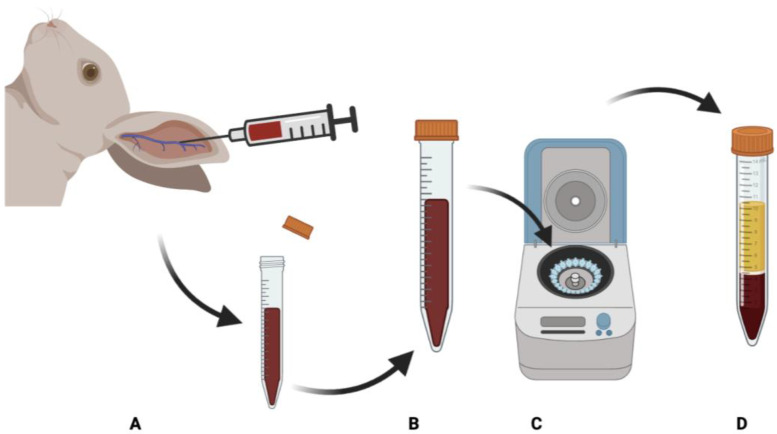
(**A**) Ear vein puncture in the rabbit using a syringe with a needle ranging between 21 to 23G; (**B**) Blood placed in a tube/falcon (without additives); (**C**) The tube is then quickly placed in the centrifuge, following protocols for PRF production, rpm, time, and RCF; (**D**) PRF is produced after following the previous steps.

The ear vein is a less-invasive method for collecting small volumes of blood from rabbits (Figure 3). This method is relatively easy, making it a preferred option for many researchers (Table 2). The rabbit’s ear usually is warmed to dilate the veins to collect the blood. The ear vein is the most common area used by researchers to produce PRF (Table 2). However, this area does not offer a large amount of blood in a favorable flow to produce the PRF membrane. Therefore, it should not be considered the first area of election to produce more than one or a larger PRF membrane.

Intracardiac function is a more invasive method of blood collection that involves accessing the heart directly. It requires surgical skills and experience and should be performed under anesthesia. As was described for the rat model, this method allows for the collection of larger volumes of blood and is more suitable for extensive manipulation of the blood sample.

**Figure 4:**
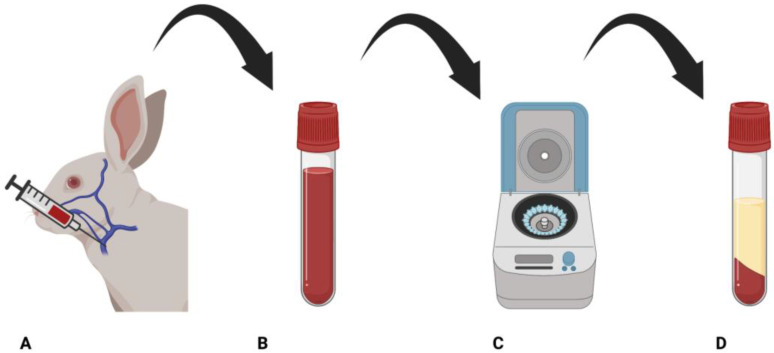
(**A**) The jugular vein puncture in the rabbit’s neck using a syringe with a needle ranging between 18–23G; (**B**) Blood placed in a tube/falcon (without additives); (**C**) The tube is then quickly placed in the centrifuge, following protocols for PRF production, rpm, time, and RCF; (**D**) PRF is produced after following the previous steps.

The jugular vein puncture is another method for collecting blood in rabbits that involves accessing the veins located in the neck area (Figure 4). This method is convenient to perform, and it is used for collecting larger volumes of blood and is best suited for the production of PRF and other blood concentrates. The jugular vein allows a faster and more significant amount of blood than the marginal ear vein, making it less stressful for the rabbit than a heart puncture [87].

The coagulation time for rabbit blood is around 5 to 10 min, which is similar to the coagulation time in humans. In addition, they are often considered the best animal model to study human platelets due to their similarities in platelet function and regulation [88,89,90,91,92,93,94,95]. Regarding platelet count, rabbits have a similar range of platelet numbers compared to humans, with an average platelet count ranging from 150,000 to 450,000 platelets per microliter of blood. However, there may be some differences in the size and distribution of platelets between rabbits and humans, which can impact platelet function and response to different stimuli [70,96].

In addition, comparing the number of cells in the blood, rabbits have a similar range of red and white blood cells compared to humans. And the number of cytokines and growth factors in rabbit blood can vary based on the age, sex, and strain of the rabbit [96,97]. Therefore, it is crucial to consider these factors when conducting research using rabbit blood samples [70].

Regarding coagulation time, rabbit blood has a slightly longer coagulation time than rat blood. However, both are faster than the human coagulation time.

## 7. Venipuncture of Dogs

Blood collection from dogs for experimental research involving blood concentrates is a common practice. The most commonly used methods for blood collection in dogs include venipuncture of the cephalic, saphenous (lower limb veins), or jugular veins (Table 3). The choice of the site for blood collection depends on the animal’s size, temperament, and the amount of blood needed for the study.

The coagulation time in dogs is similar to that of humans, usually taking around 2 to 10 min. The number of cells in the blood, cytokines, and growth factors in dogs is also similar to those found in humans [110,111]. However, it is important to note that there can be individual variations in these factors between dogs, as well as between different breeds and ages.

When compared to other animals used in experimental research, such as rats or rabbits, dogs have a longer coagulation time. This difference should be considered when designing studies that involve clotting factors or blood coagulation.

Regarding platelet count, dogs have a higher platelet count than humans, with an average of 200,000 to 500,000 platelets per microliter of blood [111]. Thus, this difference should also be taken into account when designing experiments that involve platelets.

## 8. Venipuncture of Pigs and Mini-Pigs

Blood collection in pigs and mini-pigs for studies in blood concentrates is a commonly used technique in experimental research [112,113,114,115,116,117]. The choice of blood collection site in pigs and mini-pigs will depend on the size and age of the animal. In PRF studies, the most commonly used areas for blood collection in pigs and mini-pigs are the precaval vein, jugular vein, and ear vein (Table 4).

Overall, pigs and mini-pigs are valuable models for experimental research. The use of pigs and mini-pigs in biomedical research can provide important insights into human health and disease. Careful consideration should be given to the choice of blood collection site to ensure minimal stress to the animal and maximum accuracy of results.

The time for pig blood coagulation is approximately 2 to 5 min, which is similar to human coagulation. This time may vary depending on the breed, age, and health status of the animal. The number of cells in the blood, the quantity of cytokines, and the growth factors in pig blood are also similar to that in humans. However, the platelet range is between 250,000 to 600,000 per microliter of blood [67].

It is worth noting that pigs are often used as animal models for studying human health and diseases. This is due to their anatomical and physiological similarity to humans and their porcine genome being three times closer to that of humans than the rat’s genome [119]. However, researchers should be cautious when handling pigs and mini-pigs for PRF studies, as their coagulation times can be faster than that of humans.

## 9. Venipuncture of Goats

Blood collection from goats for experimental research involving blood concentrates is an important practice. The most common method for blood collection in goats is through the jugular vein.

The coagulation time in goats is longer than that of humans, usually taking around 3 to 7 min. The number of cells in the blood, cytokines, and growth factors in goats are also similar to those found in humans. However, similar to dogs, there can be individual variations in these factors between goats, as well as between different breeds and ages [67,110].

When comparing platelet counts, goats exhibit a range similar to that of humans. On average, goats have between 100,000 and 500,000 platelets per microliter of blood, while humans typically possess 150,000 to 450,000 platelets per microliter of blood. This difference should also be taken into account when designing experiments that involve platelets [67].

It is important to note that goats are particularly useful as models for human research in studying blood products, particularly fibrinogen, due to the similarity of goat and human fibrinogen structures.

The number of cells, cytokines, and growth factors in goats is similar to those found in humans, and goats are particularly useful in studying fibrinogen [120,121]. The lower platelet count in goats compared to humans should be considered when designing experiments that involve platelets.

To perform dependable experimental studies on platelet concentrates, researchers must consider several vital factors that can affect the results. These factors include each animal’s age, gender, strain or breed, and overall health, as they can significantly influence platelet count, coagulation time (Table 5), and their corresponding averages. By addressing these factors, studies can more effectively ensure accurate and consistent findings.

## 10. Considerations on the Relative Centrifugal Force

Producing blood concentrates, such as PRF, requires precise adherence to protocols; one of these is accurately calculating the relative centrifugal force (RCF) or g force required for the centrifugation process. Errors in RCF calculations can significantly affect the quality and reproducibility of the final product, making it vital to ensure accurate RCF calculations in every study. Standardizing RCF values across different studies can also enhance comparability and facilitate the transfer of scientific knowledge from animal to human studies.

The RCF value is determined by using a formula that considers the rotor’s radius and the revolutions per minute (rpm). The formula is an essential component of the blood concentrate production process, and any errors in calculating the RCF value can significantly impact the final product’s quality. Therefore, it is crucial to ensure that the formula is accurately applied in every study to produce reliable and reproducible results. In addition, it is recommended to use standardized methods for determining the RCF value to enhance comparability between different studies. Overall, correctly applying the RCF formula is critical for producing blood concentrates necessary for various scientific and clinical applications [122,123,124,125,126].

**Figure 5:**
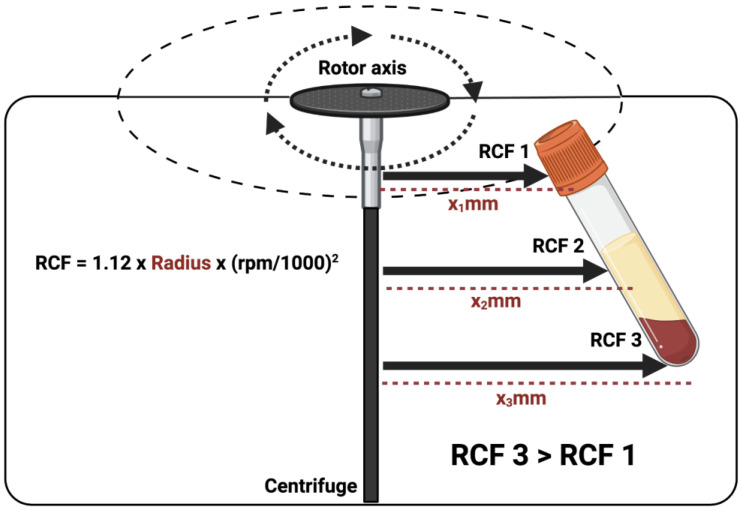
Schematic illustration of the blood centrifugation process, highlighting the minimum radius (×1 mm), average radius (×2 mm), and maximum radius (×3 mm). It is important to note that the relative centrifugal force (RCF) calculation is higher for ×3 mm than ×1 mm. Additionally, for studies that use small tubes and low amounts of animal blood and for those using a commercial centrifuge to produce PRF, the authors should consider the average radius (×2 mm) to provide the RCF in their methods.

Communication of scientific and clinical protocols is crucial to ensure the reproducibility of human studies in a standard format. The principles of translational research determine that all details of a method of study should be reported to facilitate the transfer of scientific knowledge of animal studies to humans. However, inaccuracies in reports may impair the translation of research results. The PRF protocol is an example of such inaccuracy. The protocol reports the 400× *g* centrifugation RCF but does not specify the location of the rotor in which the RCF was measured (mm). Therefore, to standardize the terminology for PRF production, it is necessary to establish an approach that focuses on the RCF obtained from the minimum radius (×1 mm), average radius (×2 mm), and maximum radius (×3 mm), as illustrated in Figure 5. In doing so, animal studies can be conducted more accurately, and the results can be applied more reliably to human studies.

Obtaining standardization in centrifugal calibration is crucial for reliable and reproducible results in the production of blood concentrate. The maximum RCF (×3 mm) is the most commonly used parameter for centrifuge calibration, as it ensures the best standardization in different models and brands.

In order to have accurate and consistent results in PRF production, it is recommended to follow the same standardized standard for human studies and use the maximum radius (×3 mm) to determine the RCF value. This value can be found in the literature [126] or obtained from the centrifuge manufacturer.

However, it is essential to note that the location of the ×3 mm may vary depending on the size and shape of the tube or hawk used for the study. Therefore, it is recommended to include a ×2 mm measurement as a standard in the study method. This measurement provides a reliable alternative to ×3 mm and can be used when the size or shape of the tube or Falcon prevents the use of ×3 mm.

In addition to the use of standard measurements, it is also crucial to report all the details of the centrifuge protocol in a standardized format to ensure reproducibility and comparability between studies. This includes reporting the rotor’s location, the centrifuge’s time and speed, and other relevant details. Following these guidelines, researchers can improve the accuracy and reliability of their results and promote the advancement of translational research in blood concentrate production.

It was important to identify the correct protocol for each centrifuge. The maximum RCF (×3 mm) is commonly used for centrifugal calibration to obtain a more reliable standardization. Consequently, in the standardization published for human studies, the maximum radius should be considered to determine the value of the RCF in the production of blood concentrate using several centrifuges available on the market. The ×3 mm information can be found in the literature or may be offered by the manufacturer.

The location of the ×3 mm mark for each study may also vary depending on the size of the tube or falcon used and the centrifuge radius. If the ×3 mm mark corresponds to a distance larger than the tube’s size, the authors should consider adding a ×2 mm mark in the study’s methods to standardize the RCF used in the protocol.

In order to ensure consistency and quality in PRF production, it is important to follow a standardized protocol. Protocol deviation can result in variations in the fibrin mesh, cell count, and growth factor content, making the scientific data obtained incomparable and potentially useless. Therefore, adhering to the established procedures for reliable and reproducible results is crucial.

## 11. Conclusions

Overall, the study concludes that standardization of animal models and techniques is essential for reducing the gap between laboratory research and clinical applications. It is crucial to report detailed information about the materials and vessel used for blood collection, the type of centrifuge used, rpm, RCF, and the maximum or average radius for the calculus, as well as the size of the tube and the amount of blood collected, to ensure reproducibility of results.

## Figures and Tables

**Table 1:** Data was collected from the PRF process using the rat model in the last five years, including one from 2023.

Author and Year	Breed	Animal Size	Number of Animals	Blood Collected (mL)	Centrifuge (RPM/min or RCF)	Donor Area
Akyildiz et al. (2018) [44]	Sprague-Dawley rats	340–380 g	23	4	3000 rpm/10 min	Intracardiac puncture
Alizadeh et al. (2023) [45]	Wistar rats	230–300 g	NR	NR	2700 rpm/12 min	Orbital sinus
Alsherif et al. (2020) [46]	Wistar rats	300–360 g	10 (PRF group)	4	2000 rpm/10 min (400 RCF)	Orbital sinus
Awadeen et al. (2020) [47]	Sprague-Dawley rats	NR	63	10	3000 rpm/10 min	Orbital sinus
da Silva et al. (2022) [11]	Wistar rats	350–450 g	24	3	2700 rpm/12 min (701 RCF-max) and 1500 rpm/14 min (216 RCF-max)	Intracardiac puncture
Demirel et al. (2018) [48]	Sprague-Dawley rats	400–450 g	28	16	3000 rpm/13 min	Intracardiac puncture
Engler-Pinto et al. (2019) [42]	Wistar rats	250–300 g	8	3.5	2700 rpm/12 min	Intracardiac puncture
Grecu et al. (2019) [49]	Wistar rats	220–420 g	35	10	1300 rpm/8 min (400 RCF)	Intracardiac puncture
Grecu et al. (2019) [50]	Wistar rats	220–420 g	35 (scarified to produce PRF)	10	1300 rpm/8 min (400 RCF)	Intracardiac puncture
Huang et al. (2020) [23]	Sprague-Dawley rats	180–220 g	24 (sacrificed to produce PRF)	5	400 g/10 min	NR
Jamalpour et al. (2022) [51]	Wistar rats	300–350 g	60	2	1500 rpm/14 min and 2700 rpm/12 min	Orbital sinus
Mirhaj et al. (2022) [52]	Wistar rats	250–300 g	3 (PRF group)	NR	2700 rpm/12 min	Orbital sinus
Mourad et al. (2022) [53]	Wistar rats	250–300 g	30	2	3000 rpm/10 min	Tail vein
Neves-Atti et al. (2022) [54]	Spontaneously hypertensive rats	250 g	40	6	3000 rpm/10 min	Intracardiac puncture
Nica et al. (2019) [55]	Wistar rats	460–550 g	40	9 to 12	450 g/12 min	Intracardiac puncture
Nugraha et al. (2018) [56]	Wistar rats	250 g	36	1.5	3000 rpm/10 min	Intracardiac puncture
Özçay et al. (2018) [22]	Sprague-Dawley rats	250–300 g	40	1	3000 rpm/10 min	Intracardiac puncture
Özçay et al. (2020) [40]	Sprague-Dawley rats	250–300 g	10 (PRF group)	1	3000 rpm/10 min	Intracardiac puncture
Padilha et al. (2019) [57]	Wistar rats	300–400 g	1 animal scarified per group	9	3000 rpm/12 min	Intracardiac puncture
Rady et al. (2022) [19]	Wistar rats	175–200 g	36	2	3000 rpm/10 min	Tail vein
Silveira et al. (2022) [58]	Wistar rats	320 g	54 (2 animals scarified for PRF)	10	2700 rpm/12 min (857 RCF max)	Intracardiac puncture
Sumida et al. (2019) [59]	Wistar rats	400–450 g	23	6	890 g/13 min	Intracardiac puncture
Tavakoli et al. (2022) [60]	Wistar rats	400 g	NR	6	1500 rpm/14 min	Orbital sinus
Tayşi et al. (2018) [61]	Sprague-Dawley rats	240–260 g	60 (including a sacrigication group)	10 to 15	3000 rpm/10 min (400 RCF)	Intracardiac puncture
Torul et al. (2018) [39]	Wistar rats	200–250 g	30	2	3000 rpm/10 min (400 RCF)	Tail vein
Vares et al. (2021) [62]	Wistar rats	250–280 g	32	2	3000 rpm/12 min (400 RCF)	Intracardiac puncture
Wang et al. (2021) [41]	Sprague-Dawley rats	NR	30	5	400 RCF/10 min	Abdominal aortic
Zhang et al. (2019) [63]	Sprague-Dawley rats	210–310 g	20	5	3000 rpm/10 min	Abdominal aortic
Zhang et al. (2022) [64]	Sprague-Dawley rats	250–300 g	32	3.5	600 RCF	Intracardiac puncture

RCF = relative centrifugal force; NR = not reported.

**Table 2:** Data was collected from the PRF process using the rabbit model in the last three years.

Author and Year	Breed	Animal Size	Number of Animals	Blood Collected (mL)	Centrifuge (RPM/min or RCF)	Donor Area
Choi et al. (2021) [77]	New Zealand	2.5–3 kg	33	5	2700 rpm/12 min	NR
Damayanti et al. (2022) [78]	New Zealand	3–4 kg	18	3	3200 rpm/10 min	Ear
Dereli-Can et al. (2020) [79]	New Zealand	3.0–3.5 kg	45	5	2700 rpm/12 min	Femoral vein
Karayürek et al. (2019) [80]	New Zealand	2.6–3.9 kg	28	8	3000 rpm/10 min	Ear
Kim et al. (2021) [81]	New Zealand	3.0–4.0 kg	12	10	3000 rpm/10 min	Ear
Kinoshita et al. (2021) [82]	New Zealand	3.5–4.2 kg	18	10	2400–3000 rpm/13 min	NR
Kızıldağ et al. (2020) [83]	New Zealand	3.0–3.5 kg	18	5	2700 rpm/12 min	Ear
Koyanagi et al. (2022) [74]	New Zealand	3.5–4.0 kg	5	2.5	700 RCF/12 min	Ear
Li et al. (2022) [73]	New Zealand	2 ± 0.2 kg	52	5	2000 RCF/5 min	Intracardiac puncture
Liu et al. (2021) [84]	New Zealand	3–4 kg	12	10	3000 rpm/10 min	Ear
Liu et al. (2019) [72]	New Zealand	2.8 and 4 kg	12	5	3000 rpm/10 min	Ear
Mogharehabed et al. (2022) [76]	New Zealand	1.5 kg	20	9	2700 rpm (408 RCF)/12 min	NR
Mu et al. (2020) [71]	New Zealand	3.0–3.5 kg	16	10	700 rpm/3 min	NR
Mudalal et al. (2019) [85]	New Zealand	3.0–3.5 kg	12	10	3000 rpm (1278 RCF)/12 min	NR
Rezuc et al. (2020) [86]	New Zealand	2 kg	12	8	3000 rpm/400 RCF/10 min	Ear
Salih et al. (2018) [87]	New Zealand	1.5–2 kg	20	3	3000 rpm/10 min	Intracardiac puncture
Şentürk et al. (2020) [14]	New Zealand	2.0–3.0 kg	27	10	3500 rpm/15 min	Ear
Shanei et al. (2022) [15]	New Zealand	2.5–3 kg	5	5	2700 rpm/8 min	Ear
Taufik et al. (2023) [16]	New Zealand	2.0–3.5 kg	15	10	3000 rpm/10 min	Ear
Wang et al. (2022) [17]	New Zealand	3–3.5 kg	10	10	3000 rpm/12 min	Ear
Wong et al. (2021) [88]	New Zealand	2–2.5 kg	24	8	2700 rpm/10 min	Ear
Zalama et al. (2022) [18]	New Zealand	N/D	30	NR	NR	NR
Zhang et al. (2023) [75]	New Zealand	3.5 ± 0.5 kg	9	5	1300 rpm/14 min	NR

RCF = relative centrifugal force; NR = not reported.

**Table 3:** Data was collected from the PRF process using the dog model.

Author and Year	Breed	Animal Size	Number of Animals	Blood Collected (mL)	Centrifuge (RPM/min or RCF)	Donor Area
Alenazy et al. (2021) [95]	Mixed breed dog	NR	4	10	3000 rpm/10 min	NR
Anwar et al. (2022) [96]	Mixed breed dog	9–14 kg	9	20	2500 rpm/15 min	NR
Benalcázar et al. (2022) [97]	Beagle dog	NR	13	18	2700 rpm/12 min	NR
El Halaby et al. (2020) [98]	Mixed breed dog	NR	9	20	3000 rpm/10 min	Right cephalic vein
Jeong et al. (2013) [99]	NR	10–15 kg	6	10	400 RCF/10 min	NR
Ji et al. (2015) [100]	NR	12–15 kg	NR	5	3000 rpm/5 min	Lower limb vein
Kazemi et al. (2014) [101]	Mixed breed dog	20–30 kg	12	20	3000 rpm/10 min	Jugular vein
Kazemi et al. (2017) [102]	Mixed breed dog	18–40 kg	12	20	3000 rpm/10 min	Jugular vein
Mohammed et al. (2021) [103]	Mixed breed dog	18–23 kg	8	5	400 RCF/10 min	Lower limb vein
Neiva et al. (2016) [104]	Beagle dog	NR	8	NR	2700 rpm/12 min	NR
Park et al. (2022) [105]	Beagle dog	NR	7	NR	408 RCF—time NR	NR
Park et al. (2016) [106]	NR	15 kg	6	10	3000 rpm/12 min	NR
Park et al. (2023) [107]	Beagle dog	15 kg	7	20	1300 rpm/8 min	Jugular vein
Xuan et al. (2014) [108]	Mixed breed dog	15–20 kg	6	20	2400 rpm/10 min	NR
Zhou et al. (2017) [109]	Beagle dog	NR	3	10	NR	Antecubital vein

RCF = relative centrifugal force; NR = not reported.

**Table 4:** Data was collected from the PRF process using the pig model.

Author and Year	Breed	Animal Size	Number of Animals	Blood Collected (mL)	Centrifuge (RPM/min or RCF)	Donor Area
Chen et al. (2014) [113]	Mini-pig	25–30 kg	20	8	3000 rpm/10 min	Superior vena cava
Li et al. (2013) [116]	Pig	NR	NR	10	2100 rpm/12 min	Precaval vein
Li et al. (2014) [117]	Pig	NR	NR	10	2100 rpm/12 min	Precaval vein
Sheu et al. (2017) [118]	Mini-pig	21.8 kg	6	8	3000 rpm/10 min	Right jugular vein
Tsai et al. (2019) [114]	Mini-pig	26.6 ± 4.1 kg	6	40	1300 g/15 min	Internal jugular vein
Yang et al. (2012) [115]	Pig	6.8–11.2 kg	21	8	3000 rpm/10 min	Right jugular vein
Yilmaz et al. (2014) [112]	Pig	60 ± 5 kg	3	10	400 RCF/10 min	Ear vein

RCF = relative centrifugal force; NR = Not reported.

**Table 5:** Comparison of platelet count and coagulation time between humans and various animal models: an overview.

Animal	Platelet Count (Platelets/µL)	Coagulation Time (Min)
Human	150,000–450,000	5 to 10
Rat	600,000–1,500,000	2 to 5
Rabbit	150,000–450,000	5 to 10
Dog	200,000–500,000	2 to 10
Mini-pig	250,000–600,000	2 to 5
Goat	100,000–500,000	3 to 7

## Data Availability

Not applicable.
